# Urinary antimicrobial peptides and cytokines as biomarkers for recurrent urinary tract infection in children and adolescents

**DOI:** 10.3389/fimmu.2026.1838231

**Published:** 2026-06-15

**Authors:** Guillermo Yepes, Hancong Chloe Tang, Natalie Holdsworth, Kristin Salamon, Laura Schwartz, Christina Ching, Steve Rust, John David Spencer

**Affiliations:** 1 Division of Infectious Diseases, Fellowship Training Program, Nationwide Children’s Hospital, Columbus, OH, United States; 2 Data Science Office, Nationwide Children’s Hospital, Columbus, OH, United States; 3 Kidney and Urinary Tract Center, The Abigail Wexner Research Institute at Nationwide Children’s Hospital, Columbus, OH, United States; 4 Ohio University Heritage College of Osteopathic Medicine, Athens, OH, United States; 5 Division of Nephrology and Hypertension, Nationwide Children’s Hospital, Columbus, OH, United States; 6 The Ohio State University College of Medicine, Columbus, OH, United States; 7 Department of Pediatric Urology, Nationwide Children’s Hospital, Columbus, OH, United States

**Keywords:** antimicrobial peptides, cytokines, pediatrics, regression model, urinary tract infection

## Abstract

**Background:**

Recurrent urinary tract infection (rUTI) is a major diagnostic and clinical challenge. Dysregulated innate immune responses, including antimicrobial peptides and cytokines, may underlie UTI susceptibility. This study investigates whether urinary concentrations of antimicrobial peptides and cytokines differ in children and adolescents with a history of rUTI and whether they can accurately classify rUTI status.

**Methods:**

In this single-center cross-sectional study, urine samples were collected from asymptomatic girls and adolescent females with a history of rUTI and age-matched controls recruited at Nationwide Children’s Hospital (Columbus, Ohio, USA). Concentrations of antimicrobial peptides (alpha-defensins 1-3, beta-defensin 1, cathelicidin, secretory leukocyte protease inhibitor, lipocalin 2, and ribonuclease 7) and cytokines (interleukin-1 beta, interleukin-6, interleukin-8, and tumor necrosis factor alpha) were quantified using enzyme-linked immunosorbent assays. A logistic regression model with variable selection was developed to classify rUTI participants based on urinary biomarkers and clinical factors.

**Findings:**

Between 2019 and 2022, urine samples were analyzed from 42 participants with rUTI and 37 healthy controls without UTI. Compared to controls, participants with rUTI had lower concentrations of beta-defensin 1, cathelicidin, and ribonuclease 7, and higher concentrations of alpha-defensins 1-3, lipocalin 2, and secretory leukocyte protease inhibitor. Cytokine concentrations, including interleukin-1 beta, interleukin-6, interleukin-8, and tumor necrosis factor alpha, were elevated in the rUTI group. A multivariable classification model integrating urinary biomarkers with clinical features demonstrated high discriminatory performance with an area under the receiver operating curve of 0.97 and a prevalence-adjusted area under the precision-recall curve of 0.94.

**Interpretation:**

Girls and adolescent females with rUTI exhibit a distinct urinary immune profile characterized by dysregulated antimicrobial peptides and elevated proinflammatory cytokines. A model integrating these biomarkers with clinical features accurately classified rUTI status, supporting their potential utility as biomarkers for identifying youth with rUTI.

## Introduction

Urinary tract infections (UTI) are among the most common bacterial infections in females across the lifespan – contributing to substantial morbidity and healthcare utilization worldwide (1, 2). Although most individuals experience sporadic infections, up to one-third develop recurrent UTI (rUTI) – defined here as ≥2 febrile or symptomatic episodes between 30 days and 24 months after the initial diagnosis. rUTI is associated with repeated antibiotic exposure, compromised kidney function, psychological distress, and reduced quality of life (2–4). Despite its prevalence and impact, clinicians lack tools to reliably identify which patients will progress from isolated to recurrent infections.

In pediatric and adolescent populations, strategies to reduce rUTI include management of bowel and bladder dysfunction, circumcision, screening for congenital kidney and urinary tract anomalies, and evaluation of sexual activity (2). Although these interventions can lower UTI incidence, they only partially explain recurrence risk. Thus, there is a critical need for biological markers that improve risk stratification beyond these clinical risk factors, enabling clinicians to identify patients most likely to develop rUTI.

Antimicrobial peptides (AMPs) and cytokines are key innate immune mediators involved in cystitis and pyelonephritis defense that show promise as UTI biomarkers (5). AMPs such as defensins, cathelicidin, ribonucleases (e.g., RNase 7), secretory leukocyte protease inhibitor (SLPI), and metal binding proteins like lipocalin 2 are produced by the urothelium of the lower urinary tract, kidney tubules, and infiltrating immune cells (6–14). Thus, urinary concentrations likely reflect combined epithelial and leukocyte contributions that may vary with inflammatory state. They exert direct antimicrobial activity as well as immunomodulatory beyond their direct antimicrobial activity, and impaired production has been linked to increased UTI susceptibility (6, 15, 16). Cytokines including interleukin 1-beta (IL-1β), IL-6, IL-8, and tumor necrosis factor alpha (TNFα) amplify local inflammation and recruit leukocytes to eradicate uropathogens. Excessive or prolonged cytokine responses can promote kidney injury, compromise the bladder barrier, alter bacterial virulence traits, and enhance infection risk (14, 17–23). While increased AMP and cytokine expression has been demonstrated during acute UTI, their expression with rUTI is poorly characterized (2, 6).

Here, we evaluate urinary AMPs and cytokines with implicated in UTI pathogenesis in asymptomatic girls and adolescent females with a rUTI history as well as UTI-naïve controls. We integrate the concentrations of these immune markers with clinical variables using logistic regression to define signatures that distinguish rUTI status. Our findings identify a distinct urinary immune signature in participants with rUTI and demonstrate strong discriminatory performance in classifying youth with recurrent infections. These insights advance our understanding of host defense in rUTI and highlight biomarker candidates that may inform future diagnostic and prevention strategies in pediatric UTI.

## Materials and methods

### Study design and participants

In this single-center cross-sectional study, participants were recruited between 2019 and 2022 from primary care, nephrology, and urology clinics at Nationwide Children’s Hospital (Columbus, OH, USA). This study was conducted in accordance with the principles of the World Medical Association’s Declaration of Helsinki, and the study protocol was approved by Nationwide Children’s Institutional Review Board. Informed written consent was obtained from all patients. For participants under 18 years of age, written parental/guardian consent and patient assent were obtained. Because UTI occurs more frequently in females, enrollment was limited to this population. Urine samples were obtained from two groups: individuals without UTI (controls) and participants with a history of rUTI.

Healthy controls were recruited from primary care clinics during routine well-child appointments. These participants had no history of UTI and were asymptomatic at the time of urine collection. A dipstick urinalysis was performed on all control urine samples. Urine cultures were not routinely obtained in this group as part of clinical care. Participants with rUTI were recruited from nephrology and urology clinics. rUTI was defined as ≥2 febrile or symptomatic, culture-confirmed UTIs occurring between 30 days and 24 months following an initial infection. Urine samples in the rUTI cohort were obtained during routine outpatient visits when participants were asymptomatic and without clinical evidence of an active infection. At the time of sample collection, both dipstick urinalysis and urine culture were performed. All cultures obtained at enrollment were negative. Prior UTIs were defined according to American Academy of Pediatrics clinical practice guidelines, with culture confirmation when available, and supplemented by clinician documentation when prior cultures were not obtainable (24). Urine microscopy was not performed in either cohort. Study participants did not receive antibiotic therapy within one month prior to urine collection.

Exclusion criteria included pregnancy, institutionalization, known immunodeficiency, use of immunosuppressive medications, malignancy, chronic antibiotic use or antibiotic treatment within 1 month of enrollment, impaired kidney function, and urinary tract anomalies (including hydronephrosis, solitary kidney, renal dysplasia, cystic kidney disease, urinary tract obstruction, or neurogenic bladder, dilating vesicoureteral reflux).

### Procedures

Clinical and demographic data were extracted from the electronic health record. Detailed information on all participants is outlined in **Supplementary Table 1, 2**. Free-void urine samples were collected from all participants using AssayAssure collection tubes (Thermo-Fisher, Waltham, MA, USA). Samples were centrifuged and urine supernatants were stored at -80°C until analysis.

Commercial assays quantified urine concentrations of α-defensins 1-3 (Hycult Biotech, Plymouth Meeting, PA, USA), β-defensin 1 (PeproTech, Cranbury, NJ, USA), cathelicidin (Hycult), lipocalin 2 (Abcam, Cambridge, United Kingdom), RNase 7 (Hycult), and SLPI (Abcam). Assays were performed following manufacturer’s instructions. Urinary cytokines (IL-1β, IL-6, IL-8, and TNFα) were measured using the multiplex V-Plex Viral Panel 1 (Meso Scale Diagnostics, Rockville, Maryland, USA). Urine creatinine was also measured (Oxford Biomedical Research, Rochester Hills, MI, USA).

### Statistical analysis

The primary objective was to compare urinary AMP and cytokine concentrations between control and rUTI participants. The secondary objective was to evaluate whether urinary biomarkers and clinical variables classify rUTI status. Cytokine concentrations were log10-transformed to improve normality. AMP concentrations were reported in absolute concentrations to preserve interpretability. Continuous variables were assessed for normality with the D’Agostino-Pearson Omnibus or Shapiro-Wilk normality test, with normality defined as a *P-*value > 0.05. Group comparisons were performed using a Student’s *t*-test or ANOVA for normally distributed data and the Mann-Whitney *U* or Kruskal-Wallis tests for nonparametric data.

To evaluate whether urinary biomarkers could distinguish rUTI from controls, a logistic regression model with least absolute shrinkage and selection operator (LASSO) regularization was developed using urinary biomarkers and clinical covariates. Because this was a cross-sectional study, the model was developed to classify rUTI status rather than future infection risk. Candidate variables included all AMPs, log10-transformed cytokines, urine creatinine, and clinical metrics including age at sample collection, body mass index percentile, and leukocyte esterase (LE). Model training used 10 repetitions of 10-fold stratified cross-validation to identify variables retained in the final model while reducing overfitting. Final model coefficients were obtained by fitting the LASSO model to the full dataset with the best hyperparameter selected by repeated cross-validation.

Model performance and uncertainty were estimated using bootstrap resampling (1,000 iterations), with each bootstrap sample undergoing ten repetitions of 10-fold stratified cross-validation using the best hyperparameter value. Performance metrics included area under the receiver operating characteristic curve (AUROC), area under the precision-recall curve (AUPRC), sensitivity, specificity, accuracy and positive predictive value (PPV). PPV and accuracy were calculated using the historical prevalence of rUTI in the target population (25%) rather than the prevalence in the study data set (2). PPV and sensitivity were plotted as a function of the percentage of individuals intervened to illustrate performance across operational thresholds. All statistical analyses were performed in R (v4.4.2) using the packages *rsample, glmnet, caret, pROC, precrec*, and *boot*.

### Role of the funding source

The NIH had no role in study design, data collection, data analysis, data interpretation, or writing of the report.

## Results

A total of 79 toilet-trained girls and adolescent females were included in the study, including 42 with a history of rUTI and 37 controls with no UTI history (**Table 1**; **Supplementary Table 1**). Controls had no chronic medical conditions other than hypertension (*n* = 4) or seasonal allergies (*n* = 3). In the rUTI group, six patients underwent a voiding cystourethrogram and all demonstrated grade II vesicoureteral reflux or less. Children with rUTI had a median of four prior infections, with uropathogenic *E. coli* (UPEC) being the most common uropathogen (**Supplementary Table 2**).

**Table 1:** Subject demographics and clinical characteristics.

Variable	Control (*n* = 37)	rUTI (*n* = 42)	*P*-value
Age at enrollment (years)	11.47 (7.21 – 15.01)	8.56 (6.46 – 12.64)	0.14
Race			
White	27 (69%)	37 (88%)	0.13
Black	7 (18%)	3 (7%)	
Other	5 (13%)	2 (5%)	
Body mass index percentile	89.15 (30.32 – 97.07)	76.85 (43.07 – 93.75)	0.52
UTI History			
Prior number of UTI	N/A	4 (3-5)	N/A
Febrile UTI		1 (0-3)	
Non-febrile UTI		3 (2-4.25)	
Leukocyte esterase			
Negative/trace	35 (95%)	30 (71%)	0.02
Small	1 (2.5%)	8 (19%)	
Moderate	0 (0%)	2 (5%)	
Large	1 (2.5%)	2 (5%)	
Urine creatinine (mg/dL)	108.1 (24.44 – 169)	79.38 (34.8 – 141.2)	0.58

Values are presented as median (interquartile range) or number (percent), as appropriate. *P*-values for age at enrollment, body mass index percentile, and urine creatinine were calculated using the Mann-Whitney *U* test. *P*-values for race and leukocyte esterase were calculated using Fisher’s exact test. N/A indicates variables not applicable to the control group.

**Figure 1:**
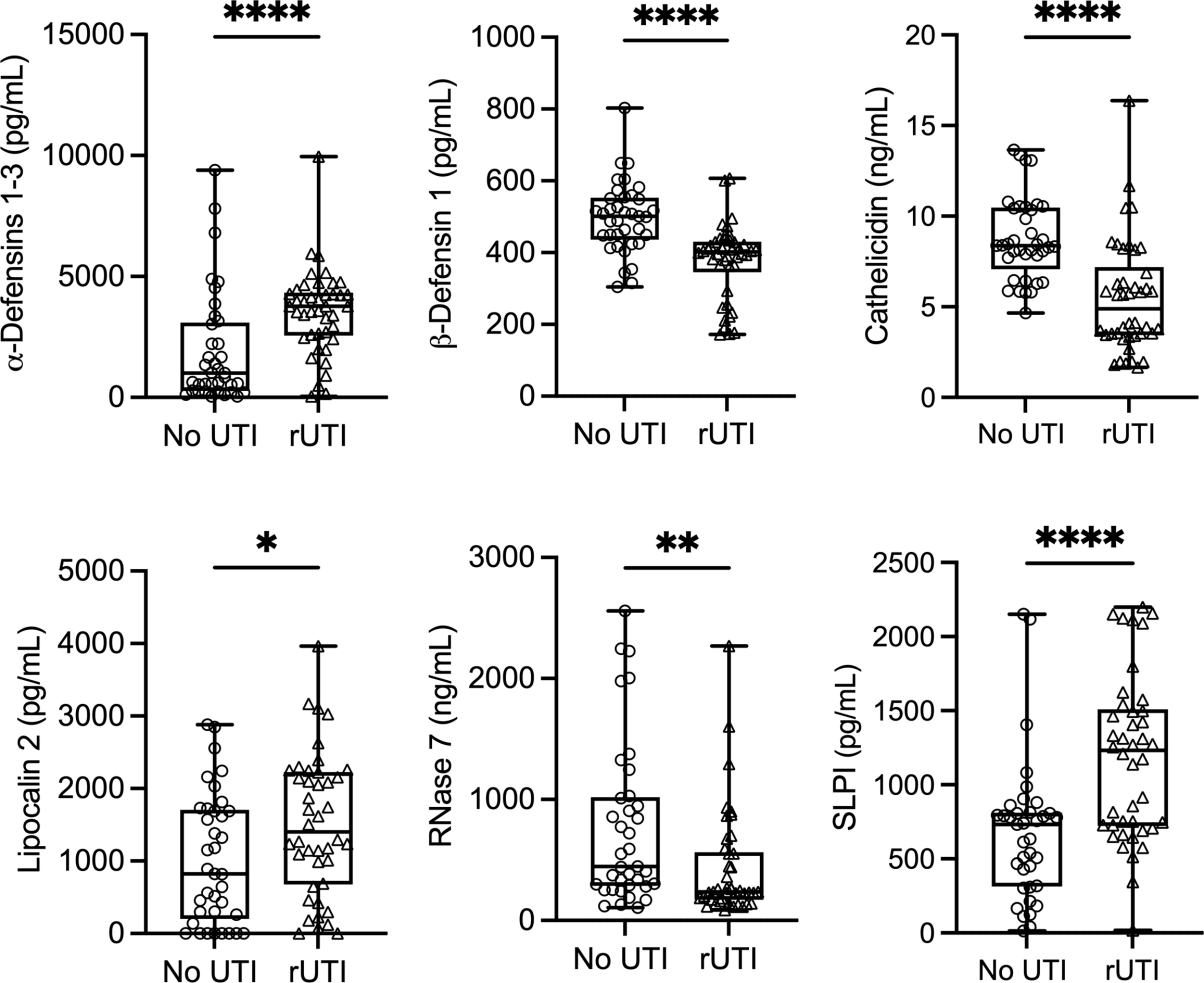
Urine antimicrobial peptides concentrations are dysregulated in girls and adolescent females with rUTI. Urinary α-defensins 1-3, β-defensin 1, cathelicidin, lipocalin 2, RNase 7, and SLPI concentrations in controls (no UTI) and youth with rUTI. Center lines show the median values, box limits indicate the 25^th^ and 75^th^ percentiles, and whiskers show the minimum to maximum range. Each symbol denotes a measurement in a different person. Asterisks denote significant *P*-values for the indicated comparisons (Mann-Whitney *U* test). *P < 0.05, **P < 0.01, and ****P < 0.0001.

Compared to controls, participants with rUTI had lower urinary concentrations of β-defensin 1 [median (interquartile range): 500.6 (436.5 – 552.8) vs. 401.8 (345.3 – 430.8) pg/mL], cathelicidin [8.37 (7.1 – 10.47) vs. 4.9 (3.45 – 7.19) ng/mL], and RNase 7 [445.7 (285.7 – 2559) vs. 236.4 (170.5 – 562.9) ng/mL]. In contrast, α-defensins 1–3 [1005 (263.8 – 3089) vs. 3765 (2559 – 4327) pg/mL], lipocalin 2 [823 (200.4– 1706) vs. 1402 (678.9 – 2227) pg/mL], and SLPI [731.5 (313.6 – 810.4) vs. 1232 (722.5 – 1509) pg/mL] were significantly elevated in the rUTI cohort (**Figure 1**). These findings demonstrate two distinct patterns of AMP dysregulation in rUTI, characterized by reduced concentrations of peptides associated with uroepithelial antimicrobial defense and increased concentrations of peptides enriched in leukocytes (6). When concentrations were normalized to urine creatinine, similar directional trends were observed, although β-defensin 1 no longer reached significance (**Supplementary Figure 1**). To evaluate potential age-related differences, analyte concentrations were stratified by pubertal age group (<8 vs ≥8 years). No consistent differences in AMPs were observed between age groups (**Supplementary Table 3**).

Across the entire cohort, AMP concentrations were stratified by LE on dipstick urinalysis (negative/trace vs. ≥ small). α-defensins 1–3 and lipocalin 2 were significantly elevated in LE-positive samples, consistent with inflammation-associated increases in these peptides (8, 12). SLPI showed a similar pattern but did not reach significance. In contrast, β-defensin 1, cathelicidin, and RNase 7 showed no correlation with LE, consistent with their classification as uroepithelial enriched peptides (**Supplementary Figure 2**) (7, 9, 10). Additionally, the number of prior UTIs showed inverse correlations with AMPs associated with uroepithelial defense and positive correlations with AMPs associated with leukocyte responses (**Supplementary Figure 3**).

**Figure 2:**
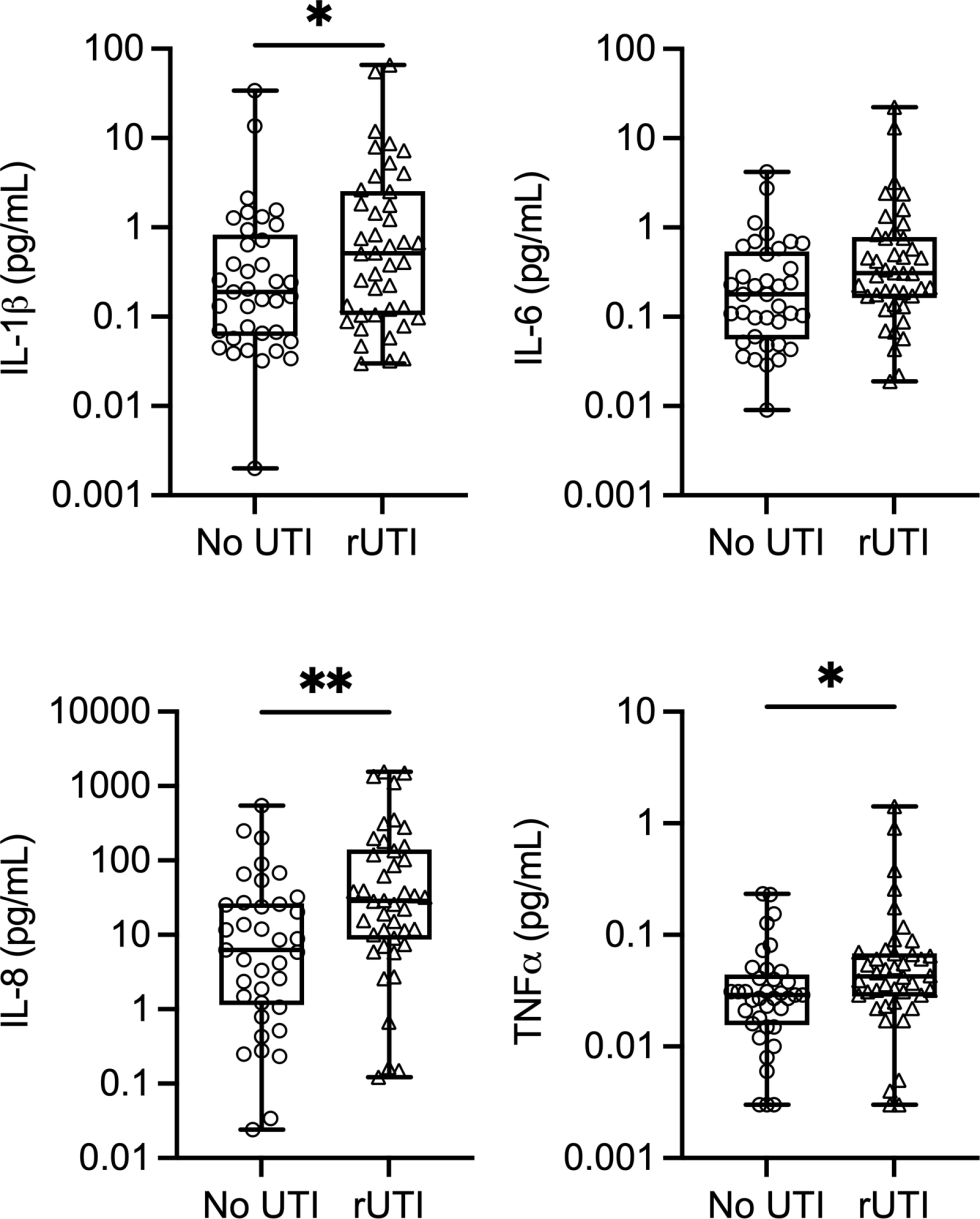
Urine cytokine concentrations are elevated in girls and adolescent females with rUTI. Urinary IL-1β, IL-6, IL-8, and TNFα concentrations in controls (no UTI) and youth with rUTI. Center lines show the median values, box limits indicate the 25^th^ and 75^th^ percentiles, and whiskers show the minimum to maximum range. Each symbol denotes a measurement in a different person. Asterisks denote significant *P*-values for the indicated comparisons (Mann-Whitney *U* test). *P < 0.05 and **P < 0.01.

Cytokine concentrations were elevated in participants with rUTI. Median IL-1β [0.18 (0.06 – 0.83) vs. 0.51 (0.1 – 2.55) pg/mL], IL-6 [0.18 (0.06 – 0.54) vs. 0.31 (0.16 – 0.78) pg/mL], IL-8 [6.25 (1.15 – 26.41) vs. 28.66 (8.66 – 140.5) pg/mL], and TNFα [0.03 (0.02 – 0.04) vs. 0.04 (0.03 – 0.07) pg/mL] concentrations were elevated in the rUTI cohort (**Figure 2**). Normalization of cytokine concentrations to urine creatinine produced similar directional trends, although statistical significance differed for some analytes compared with analyses using raw concentrations (**Supplementary Figure 4**). No consistent differences in cytokine concentrations were observed by pubertal status within either cohort (**Supplementary Table 3**).

Across the entire cohort, cytokine concentrations were further stratified by LE and examined in relation to prior number of infections. IL-1β, IL-6, IL-8, and TNFα each showed positive associations with LE, consistent with their role as inflammation-driven proteins (**Supplementary Figure 5**). In addition, cytokine concentrations showed positive associations with the number of prior UTIs, supporting a link between rUTI and sustained inflammatory signaling (**Supplementary Figure 6**).

**Figure 3:**
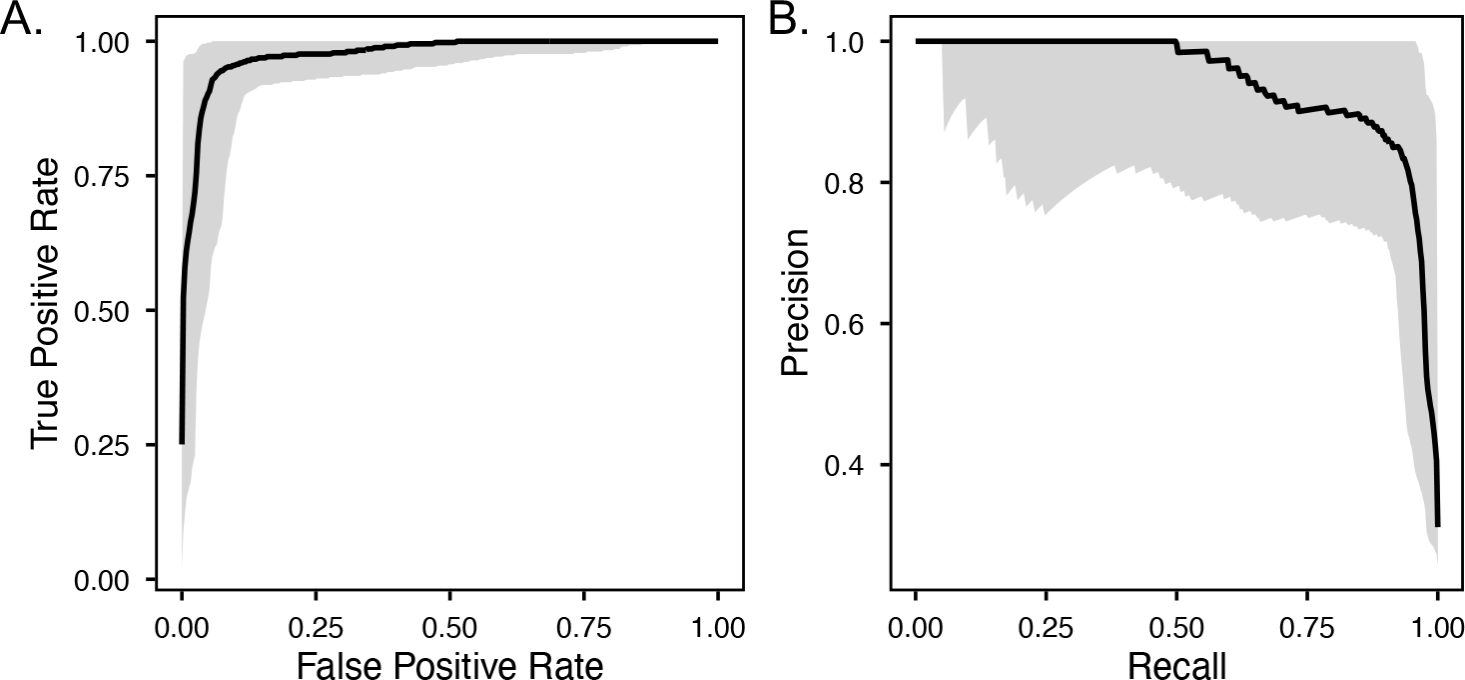
Performance of the LASSO logistic regression model for classification of rUTI. **(A)** Receiver operating characteristic (ROC) curve of the logistic regression model integrating urinary antimicrobial peptides, cytokines, and clinical variables. The solid line shows the median ROC curve from 1,000 bootstrapped resamples using repeated cross-validation. The shaded region represents the 95% confidence region derived from these resamples. **(B)** Precision-recall (PR) curve of the same model. The solid line shows the median PR curve based on 1,000 bootstrapped resamples with repeated cross-validation. The shaded region represents the 95% confidence region derived from these resamples.

Given the distinct urinary AMP and cytokine patterns observed, we next asked whether these markers could be combined with clinical covariates to classify rUTI status. All measured absolute AMP and cytokine concentrations, urine creatinine, and clinically obtained variables (age, body mass index percentile, and LE) were included as candidate variables in a LASSO logistic regression. Repeated cross-validation and bootstrap resampling were then used to select an optimal subset of variables and to estimate model performance. The final optimized model, containing only the selected variables, distinguished rUTI cases from controls with excellent discriminatory performance – achieving a median AUROC of 0.97 (95% CI: 0.94 - 0.99) and a median prevalence-adjusted AUPRC of 0.94 (95% CI: 0.82 - 0.99) (**Figures 3A, B**). We repeated modeling using AMP and cytokine concentrations normalized to urine creatinine. Model performance was modestly reduced compared with the primary analysis (median AUROC 0.93, 95% CI: 0.86 - 0.98; AUPRC 0.85, 95% CI: 0.63 - 0.98). Therefore, subsequent analyses focused on absolute concentrations.

**Figure 4:**
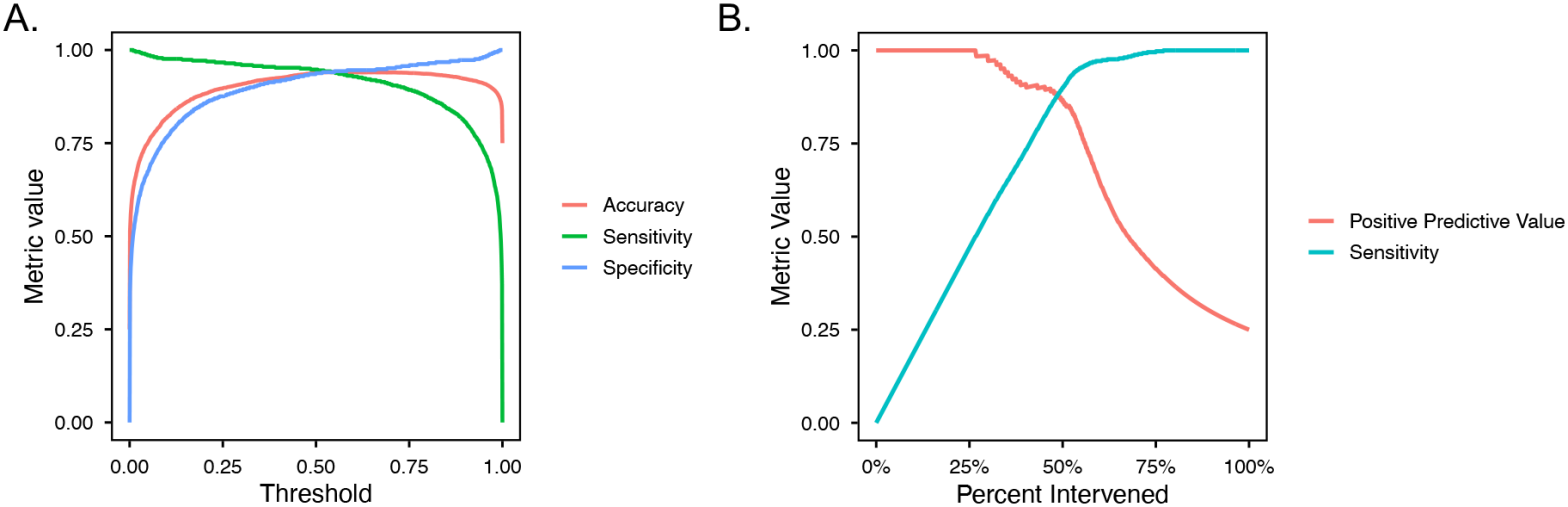
Threshold dependent performance metrics for the LASSO logistic regression model. **(A)** Sensitivity, specificity, and prevalence-weighted accuracy plotted across rUTI probability thresholds. The vertical line indicates the threshold (0.57) that maximized prevalence-weighted accuracy. **(B)** Prevalence-adjusted positive predictive value (left y-axis) and sensitivity (right y-axis) versus the percentage of individuals the model would flag for clinical action (i.e., classified as rUTI) at each risk-score threshold, based on aggregated classification probabilities from repeated cross-validation. Both curves represent median metric values from 1,000 bootstrapped samples.

Threshold analysis demonstrated stable performance across a range of decision boundaries. At the optimal threshold of 0.57 – selected to maximize prevalence-adjusted accuracy (**Figure 4A**) – the model achieved 94.1% accuracy (95% CI: 88.9 - 98.7), 93.6% sensitivity (95% CI: 87.1 - 98.8), and 94.3% specificity (95% CI: 87.8 - 99.5). At this threshold, the median PPV was 84.6% (95% CI: 71.6 - 98.4), while the negative predictive value was 97.7% (95% CI: 95.7 - 99.6). To illustrate clinical tradeoffs, PPV and sensitivity were evaluated across increasing proportions of individuals ranked by model classification score. Intervening on the top 21.9% of individuals yielded a PPV of 100% with a sensitivity of 41.7%, whereas intervening on the top 47.2% achieved a balanced PPV and sensitivity of approximately 85%, highlighting flexibility in selecting operational thresholds based on clinical priorities (**Figure 4B**).

Model feature stability was evaluated using bootstrap resampling. A subset of parameters was repeatedly retained across bootstrap iterations, including SLPI, α-defensins 1-3, LE, RNase 7, β-defensin 1, and cathelicidin. In contrast, cytokines, age, body mass index percentile, and urine creatinine were inconsistently selected across bootstrap iterations and were not retained in the final optimized model. The stable variables were refit to obtain the final model coefficients and odds ratios (**Table 2**), from which the AUROC and AUPRC values were derived. In this model, higher urinary concentrations of SLPI, α-defensins 1-3, and leukocyte esterase were associated with increased odds of rUTI, whereas higher concentrations of RNase 7, β-defensin 1, and cathelicidin were associated with reduced odds of rUTI. These findings suggest that a urinary AMP-dominant signature provides strong discriminatory power for identifying youth with rUTI.

**Table 2:** Final LASSO logistic regression model identifying variables retained in the rUTI classification model.

Feature	LASSO Logistic β	Odds ratio
SLPI (pg/mL)	0.57	1.77
α-defensins 1-3 (pg/mL)	0.46	1.58
Leukocyte esterase (positive)	0.36	1.43
RNase 7 (ng/mL)	-0.54	0.58
β-Defensin 1 (pg/mL)	-0.75	0.47
Cathelicidin (ng/mL)	-0.80	0.45

Regression coefficients (β) were obtained by fitting the LASSO logistic model to the full data set using the optimal penalty parameter selected by repeated cross-validation. Odds ratios (OR) were calculated as the exponential of the regression coefficient (exp β), representing the multiplicative change in the odds of rUTI per unit increase in each variable. Positive coefficients (OR > 1) indicate association with increased rUTI risk, while negative coefficients (OR < 1) indicate association with reduced risk.

## Discussion

Key findings from this study demonstrate that girls and adolescent females with a history of rUTI exhibit a urinary immune profile characterized by dysregulated AMPs and elevated cytokines. AMPs such as β-defensin 1, cathelicidin, and RNase 7 were suppressed, while α-defensins 1-3, lipocalin 2, and SLPI were elevated, alongside higher concentrations of IL-1β, IL-6, IL-8, and TNFα. These abnormalities suggest that impaired urothelial defenses and a heightened inflammatory milieu may predispose individuals to recurrent infections, highlighting a shift from constitutive or inducible epithelial antimicrobial defense toward a persistent inflammatory, leukocyte-associated urinary immune profile in children with rUTI. Importantly, integration of these biomarkers with clinical features in a supervised classification model identified a compact urinary signature that achieved excellent classification accuracy, supporting their potential as rUTI diagnostic tools.

Our finding that cathelicidin and RNase 7 are suppressed with rUTI supports prior preclinical and clinical studies linking these peptides to UTI defense. In mouse models, cathelicidin deletion predisposes to acute UTI, while siRNA-mediated silencing of RNase 7 enhances urothelial UPEC invasion (9, 10). In humans, pediatric and adult studies suggest that serum and urinary cathelicidin levels increase during acute UTI but decrease to below-baseline levels with UTI resolution in otherwise healthy premenopausal women, suggesting that incomplete recovery may increase recurrence vulnerability (9, 13, 25–28). Further, in a separate rUTI pediatric cohort we also found lower RNase 7 concentrations (10). Although β-defensin 1 was suppressed in our rUTI cohort, its contributions to UTI defense are less defined. *Defb1* knockout mice show increased susceptibility to *Staphylococcus* bacteriuria but not to UPEC infection (7, 29). Still, lower urinary β-defensin 1 was detected in women undergoing pelvic floor surgery with lower concentrations associated with pre-operative positive urine cultures – suggesting that β-defensin 1 deficiency may promote UTI susceptibility in specific clinical contexts (30). Prior work has highlighted the diagnostic and prognostic relevance of these AMPs in UTI (5, 6, 13). Whether reduced cathelicidin, RNase 7, or β-defensin 1 expression is a cause or consequence of rUTI remains unclear. One possibility is that rUTI-triggered inflammatory injury or repeated urothelial remodeling suppresses urothelial AMP production over time. Alternatively, individuals with intrinsically lower baseline AMP expression may have impaired mucosal defense responses that predispose them to rUTI. Given the cross-sectional nature of our study, these possibilities cannot be distinguished and justify the need for future mechanistic studies.

Leukocyte-expressed peptides were also deregulated in our rUTI cohort. α-defensins 1-3 (human neutrophil peptides), lipocalin 2 (neutrophil gelatinase-associated lipocalin), and SLPI are highly expressed in neutrophils but are also produced by kidney and bladder uroepithelial cells, indicating that their urinary levels reflect combined epithelial and leukocyte contributions (8, 11, 12). Elevated urinary α-defensin 1 (human neutrophil peptide 1) has been reported in adults with chronic pyelonephritis and correlates with leukocyte recruitment (31). Findings for lipocalin 2 are more variable. Early work noted lower urinary lipocalin 2 in children with rUTI (32). However, subsequent studies have yielded mixed results – one confirming lower lipocalin 2 concentrations in children with rUTI, while another finding no difference when comparing rUTI with culture-negative urine samples or a first-time UTI. These discrepancies may reflect differences in study design, sample sizes, disparate populations, or assay platform (Western blot vs. ELISA) (18, 32–34). SLPI, another neutrophil-associated peptide, was elevated in our rUTI cohort. In non-pregnant women, SLPI increases with uropathogen detection in individuals without a rUTI history, but this response is blunted in individuals with recurrent infection and concurrent uropathogen detection (11). Alternatively, lower urinary SLPI concentrations have been reported in adults with active infection performing self-catheterization (35). Together, these findings suggest that SLPI regulation may differ by age, infection status, bladder pathology, pathogen exposure, or recurrence phenotype. Preclinical models demonstrate that SLPI knockout mice experience higher bacterial burden and prolonged bladder inflammation after UTI, consistent with its protective role (11).

In parallel with AMP dysregulation, we observed elevated urinary IL-1β, IL-6, IL-8, and TNFα in individuals with a history of rUTI. During acute infections, UPEC activate Toll-like receptor 4 signaling, triggering cytokine and chemokine release that recruit neutrophils and other immune cells to promote inflammation and bacterial clearance. In both humans and animal models, inflammation correlates with UTI severity and recurrence (19, 36). Pediatric and adult studies show that systemic and/or urinary cytokines like IL-1β, IL-6, and IL-8 rapidly rise during UTI and may help distinguish cystitis from pyelonephritis (i.e. non-febrile versus febrile UTI) (5, 37–40). Preclinical data further demonstrate that during acute UTI in mice, elevated serum levels of granulocyte colony-stimulating factor, IL-6, and the IL-8 analog keratinocyte-derived chemokine (KC) predict chronic cystitis and subsequent infections (20). Similarly, excessive or prolonged cytokine activation can be detrimental. Experimental UTI models show that sustained IL-1β or TNFα signaling amplifies inflammation and tissue injury (19, 41–43). Our findings extend these observations by demonstrating that cytokine elevations persist in children with rUTI when sampled in the absence of acute infection. Although our cross-sectional design cannot establish causality, this pattern raises the possibility of a chronic, low-grade inflammatory state that may reduce the urothelium’s ability to neutralize uropathogens. Such sustained inflammation may arise from host genetic factors, quiescent intracellular reservoirs or biofilm-like bacterial communities, or lasting epithelial or epigenetic reprogramming – processes that have been implicated in UPEC susceptibility (19, 44). Consistent with this concept, preclinical studies demonstrate that prior UTI can sensitize the bladder, leading to persistent inflammatory signaling and epithelial remodeling even after bacterial clearance, which increases susceptibility to subsequent infections (20, 45, 46).

Importantly, our logistic regression identifies a set of features that classify rUTI status with high accuracy. These findings reinforce evidence showing that urinary immune signatures and acute phase proteins can sharpen diagnostic precision and complement clinical risk factors to improve rUTI risk stratification (5, 18, 47, 48). Additional studies in pediatric neurogenic bladder populations have similarly shown that urinary innate immune biomarkers, including lipocalin 2, may help distinguish clinically significant infection from asymptotic bacteriuria (49). Because LE differed between cohorts, it is possible that some biomarker differences reflect variation in urinary leukocyte content at the time of sampling. However, several AMPs remained strongly associated with rUTI status even when LE was included as a candidate variable in the LASSO model. These findings suggest that urinary AMP patterns capture alterations in host defense that are not fully explained by leukocyte abundance alone. Notably, although cytokines were consistently elevated in individuals with rUTI, they did not independently improve rUTI classification once AMPs were considered in multivariable modeling, suggesting that inflammatory signals may reflect downstream responses rather than primary determinants of recurrence risk.

Earlier recognition of individuals at greatest risk for rUTI has major clinical implications. Timely identification could reduce diagnostic uncertainty and enable proactive management – allowing clinicians to intensify surveillance, optimize antimicrobial prophylaxis or non-antibiotic prevention, target behavioral risk factors, or expedite decisions regarding surgical procedures to correct anatomic anomalies before they lead to more severe or repeated infections. High-risk patients might also benefit from closer follow-up after an acute UTI and early referral to subspecialty care, while lower-risk patients could avoid unnecessary antibiotics or invasive evaluations. Such targeted strategies may improve antibiotic stewardship, improve patient quality of life, reduce healthcare costs, and minimize rUTI sequelae.

While this study shows promise for developing biomarkers for identifying rUTI, several limitations should be considered. The sample size was modest, limiting power for subgroup analyses and increasing the risk of model overfitting relative to the number of variables evaluated. To mitigate this risk, we implemented 10-fold cross-validation with hyperparameter tuning restricted to the training folds, thereby reducing information leakage and ensuring that model performance was evaluated on unseen data. Nonetheless, external validation in independent cohorts is essential. Most participants were white, underscoring the need for validation in more racially and ethnically diverse populations. Because enrollment was restricted to girls and adolescent females, generalizability to males, neonates, and adults may be reduced. It is also unclear how urinary tract anomalies such as high-grade reflux or neurogenic bladder influence urinary AMP or cytokine profiles. Urine microscopy and quantitative white blood cell counts were not performed, and urine cultures were not obtained in the control samples. Although participants were clinically asymptomatic with a negative dipstick urinalysis, unrecognized asymptomatic bacteriuria cannot be excluded. Another limitation is the cross-sectional study design, which precludes causal inference regarding whether the observed alterations in AMPs and cytokines represent predisposing host factors, consequences of prior infection, or transient fluctuations in immune activity. Samples were cross-sectional, collected at single time points, and therefore do not capture possible longitudinal changes in AMP or cytokine expression during or between infections. Longitudinal urine sampling during an initial or subsequent infection, following treatment, and during antibiotic prophylaxis was not performed and warrants future investigation. In addition, molecular characterization or longitudinal strain typing of bacterial isolates was not performed, precluding distinction between recurrent infection caused by persistent versus distinct uropathogenic strains. Finally, although creatinine normalization is often used in biomarker studies, it assumes analyte concentrations scale proportionally with urine concentration, which may not hold for inflammatory peptides released from epithelial cells or infiltrating leukocytes. This consideration may be particularly relevant in pediatric populations, where concentrations vary due to differences in age, body size, muscle mass, diet, and hydration status. In our dataset, creatinine normalization attenuated or shifted several biomarker trends and reduced model performance.

In summary, this study identifies a distinct urinary immune signature in girls and adolescent females with rUTI, characterized by dysregulated AMP expression and heightened cytokine production. A discriminatory model integrating primarily AMP features and LE accurately classified rUTI status, highlighting the promise of urinary biomarkers for UTI risk stratification. Future studies need to validate these biomarkers in larger, prospective cohorts, incorporate clinical-grade assays, and test generalizability across diverse populations. Development of biomarker-based diagnostics could enable earlier identification of individuals at risk for UTI recurrence and guide targeted management strategies.

## Data Availability

The original contributions presented in the study are included in the article/**Supplementary Material**. Further inquiries can be directed to the corresponding author.
