# Supplementary material for: Urinary antimicrobial peptides and cytokines as biomarkers for recurrent urinary tract infection in children and adolescents
Source: Front Immunol. 2026 Jun 15;17:1838231. doi: 10.3389/fimmu.2026.1838231 (PMC13310754; doi:10.3389/fimmu.2026.1838231)
Supplement: Supplementary file 1 [file DataSheet1.pdf]

SUPPLEMENTAL FIGURES AND FIGURE LEGENDS

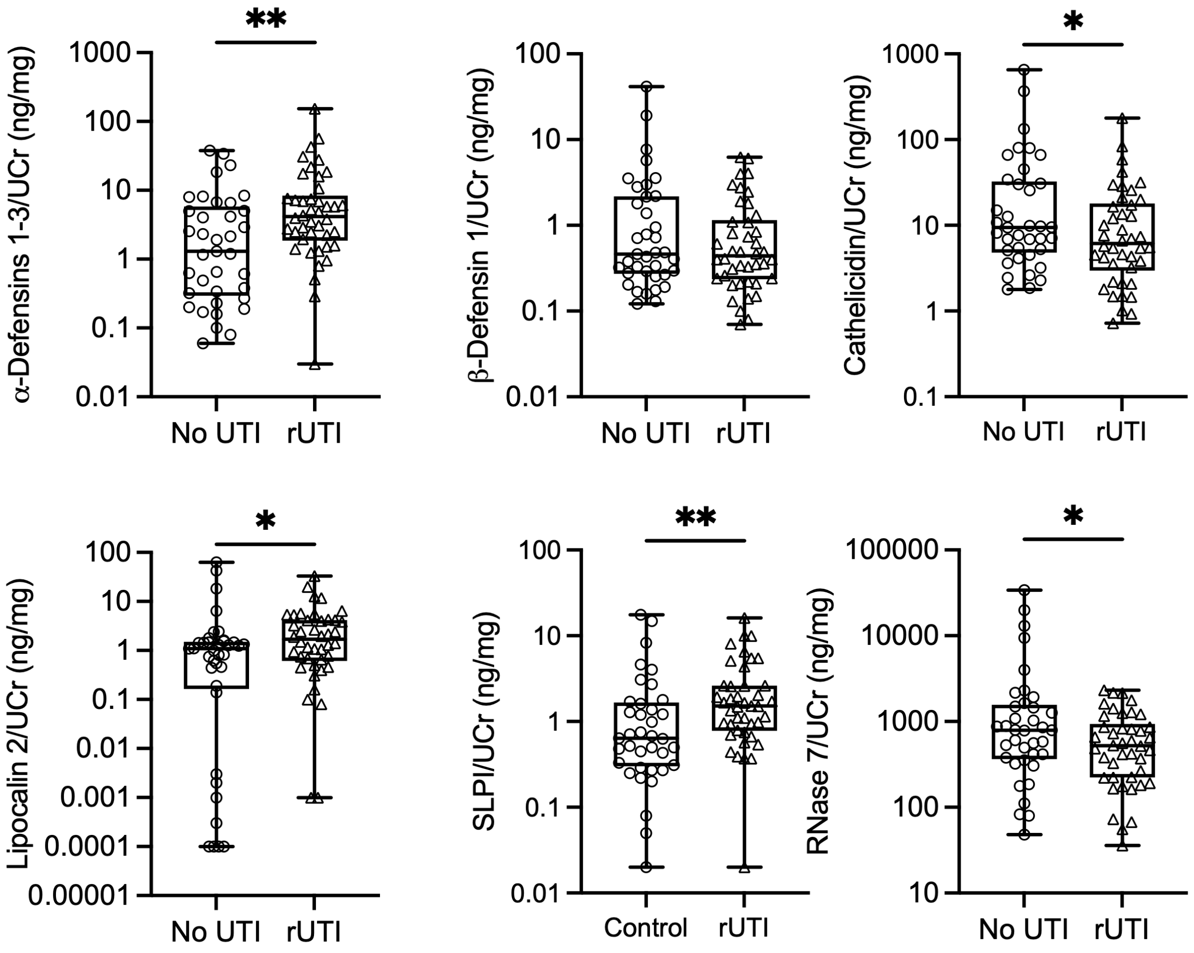

Supplemental Figure 1. Antimicrobial peptide concentrations normalized to urine creatinine. Urinary α-defensins 1-3, β-defensin 1, cathelicidin, lipocalin 2, RNase 7, and SLPI concentrations in controls (no UTI) and youth with rUTI normalized to urine creatinine (UCr). Center lines show the median values, box limits indicate the 25th and 75th percentiles, and whiskers show the minimum to maximum range. Each symbol denotes a measurement in an individual participant. Asterisks denote significant *P*-values for the indicated comparisons (Mann-Whitney *U* test). **P* <0.05, ***P* <0.01.

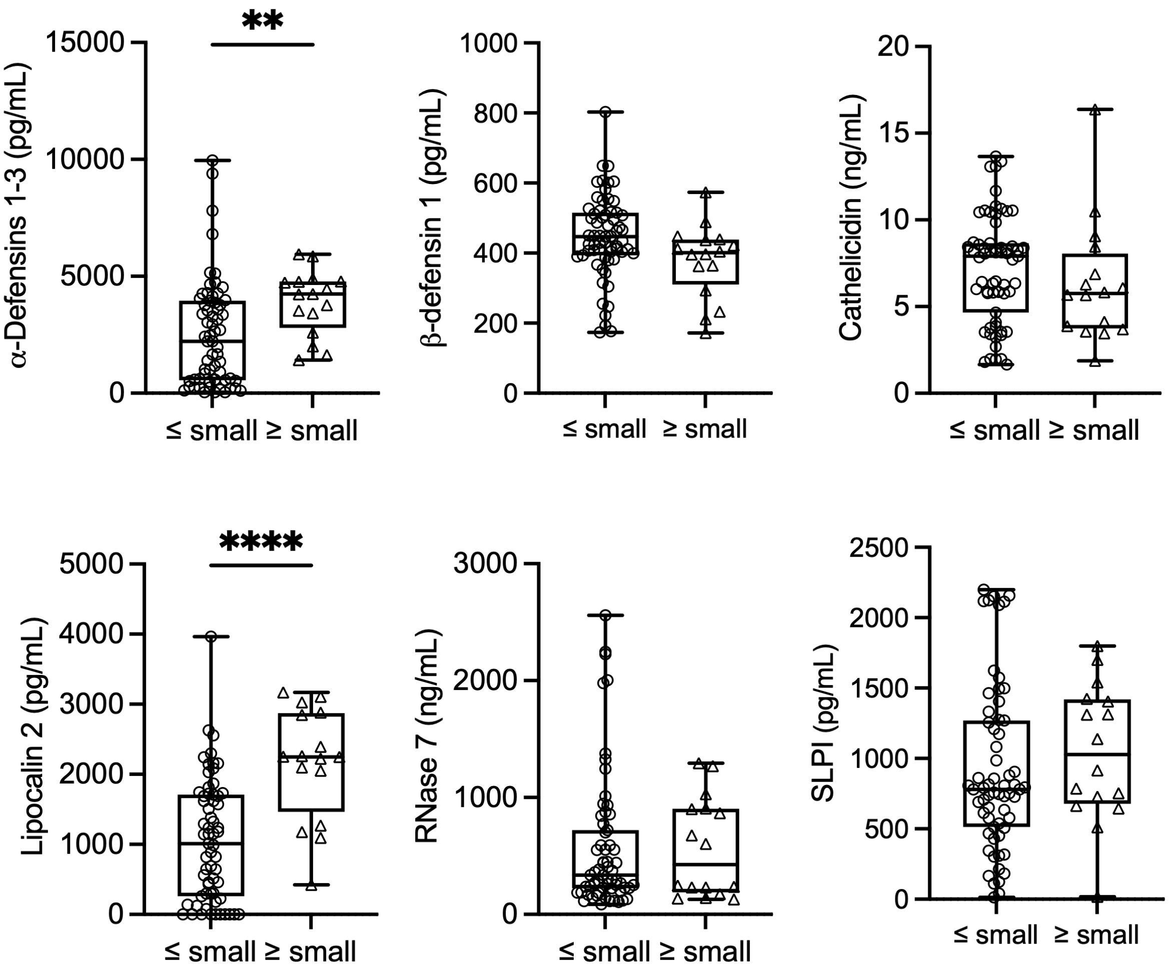

Supplemental Figure 2. Urine antimicrobial peptides stratified by urine leukocyte esterase. Urinary α-defensins 1-3, β-defensin 1, cathelcidin, lipocalin 2, RNase 7, and SLPI concentrations in all study participants grouped by urine leukocyte esterase (≤ small (negative/trace) vs. ≥ small) as measured by dipstick urinalysis. Center lines show the median values, box limits indicate the 25th and 75th percentiles, and whiskers show the minimum to maximum range. Each symbol denotes a measurement in a different person. Asterisks denote significant *P*-values for the indicated comparisons (Mann-Whitney *U* test). **P <0.01 and ****P <0.0001.

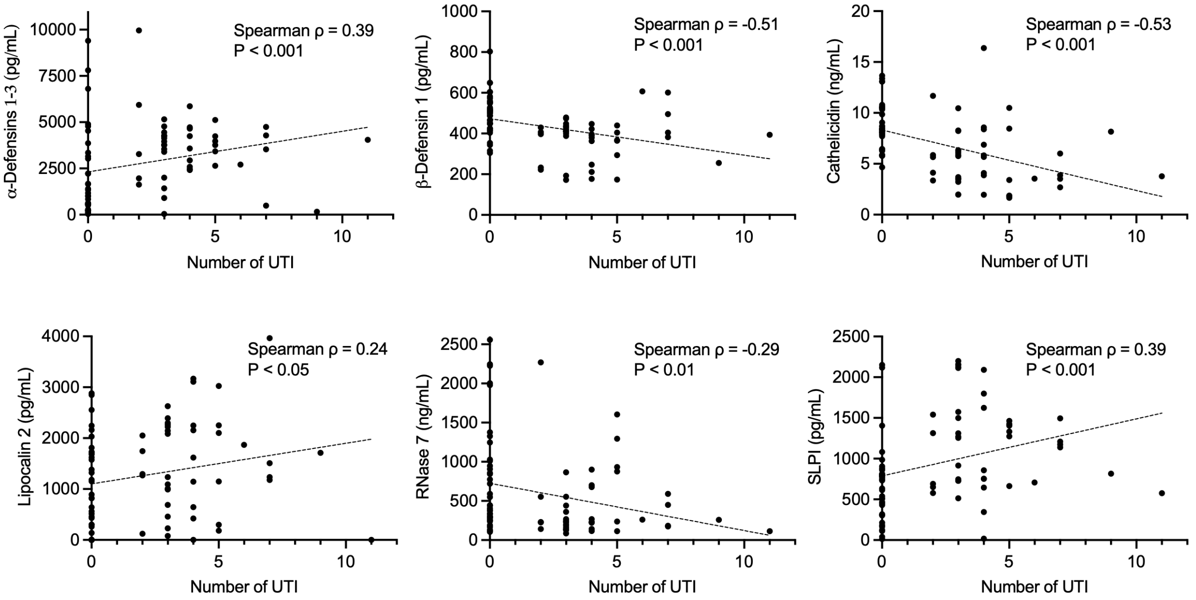

Supplemental Figure 3. Spearman correlation of urinary antimicrobial peptides with number of prior UTI. Scatter plots depict the relationship between urinary AMP concentrations and the number of prior UTIs across the entire study cohort. Spearman rank correlation coefficients (ρ) and corresponding two-tailed *P*-values are shown for each AMP. Epithelial-derived AMPs (β-defensin 1, cathelicidin, and RNase 7) demonstrate inverse correlations with UTI number, whereas leukocyte-associated peptides (α-defensins 1–3, lipocalin 2, and SLPI) show positive correlations with increasing UTI burden. Dotted lines indicate linear trend lines for visualization only.

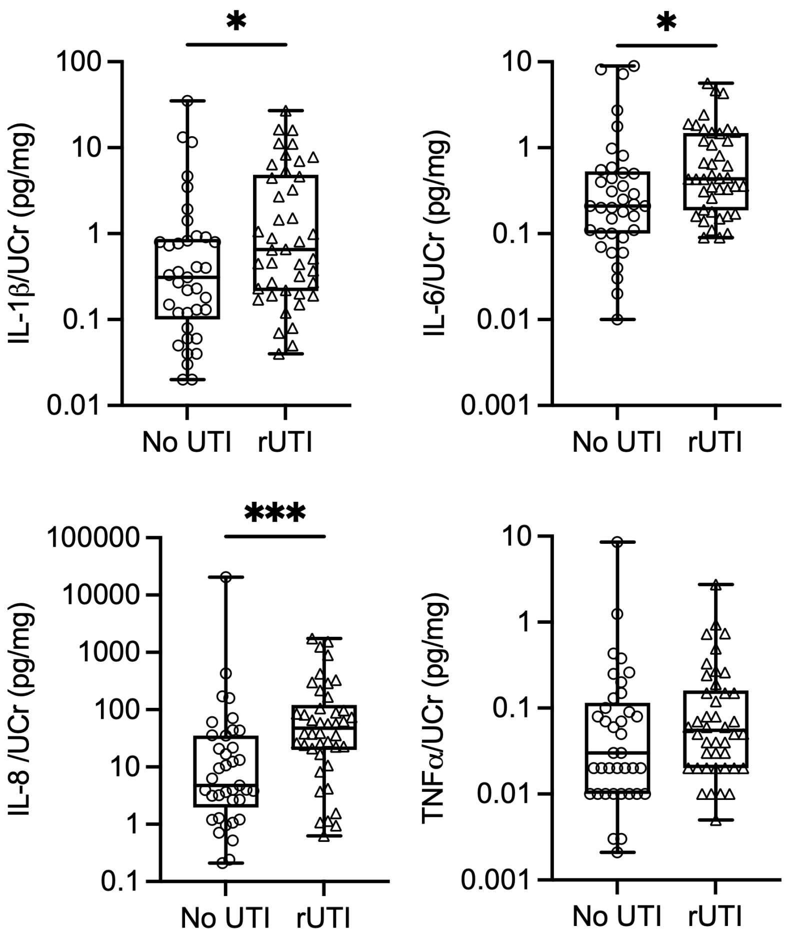

Supplemental Figure 4. Cytokine concentrations normalized to urine creatinine. Urinary IL-1β, IL-6, IL-8, and TNFα concentrations in controls (no UTI) and youth with rUTI normalized to urine creatinine (UCr). Center lines show the median values, box limits indicate the 25th and 75th percentiles, and whiskers show the minimum to maximum range. Each symbol denotes a measurement in a different person. Asterisks denote significant *P*-values for the indicated comparisons (Mann-Whitney *U* test). *P <0.05, ***P <0.001.

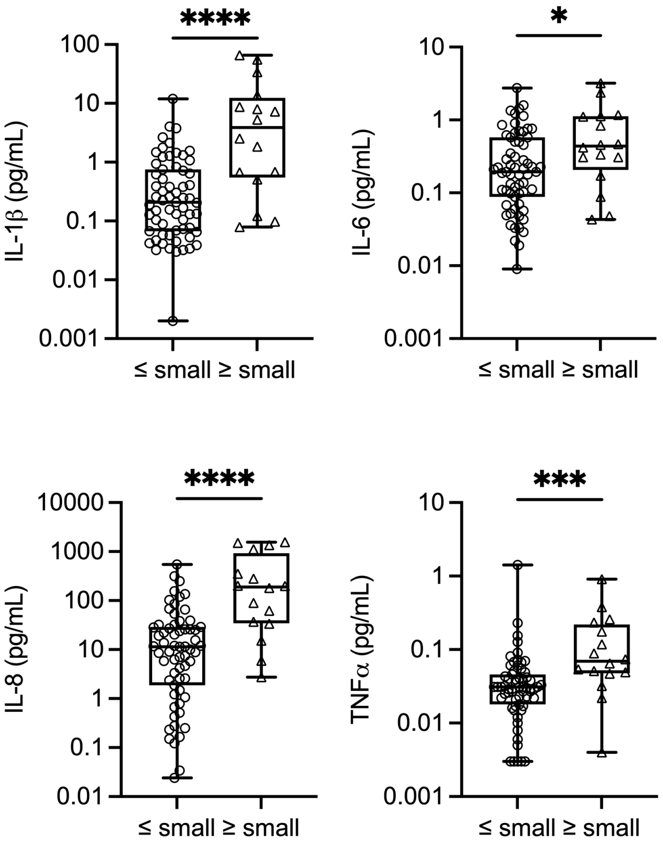

Suppemental Figure 5. Urine cytokine concentrations stratified by urine leukocyte esterase. Urinary IL-1β, IL-6, IL-8, and TNFα concentrations in all study participants grouped by urine leukocyte esterase (≤ small (negative/trace) vs. ≥ small) as measured by dipstick urinalysis. Center lines show the median values, box limits indicate the 25th and 75th percentiles, and whiskers show the minimum to maximum range. Each symbol denotes a measurement in a different person. Asterisks denote significant *P*-values for the indicated comparisons (Mann-Whitney *U* test). *P <0.05, ***P <0.001, and ****P <0.0001.

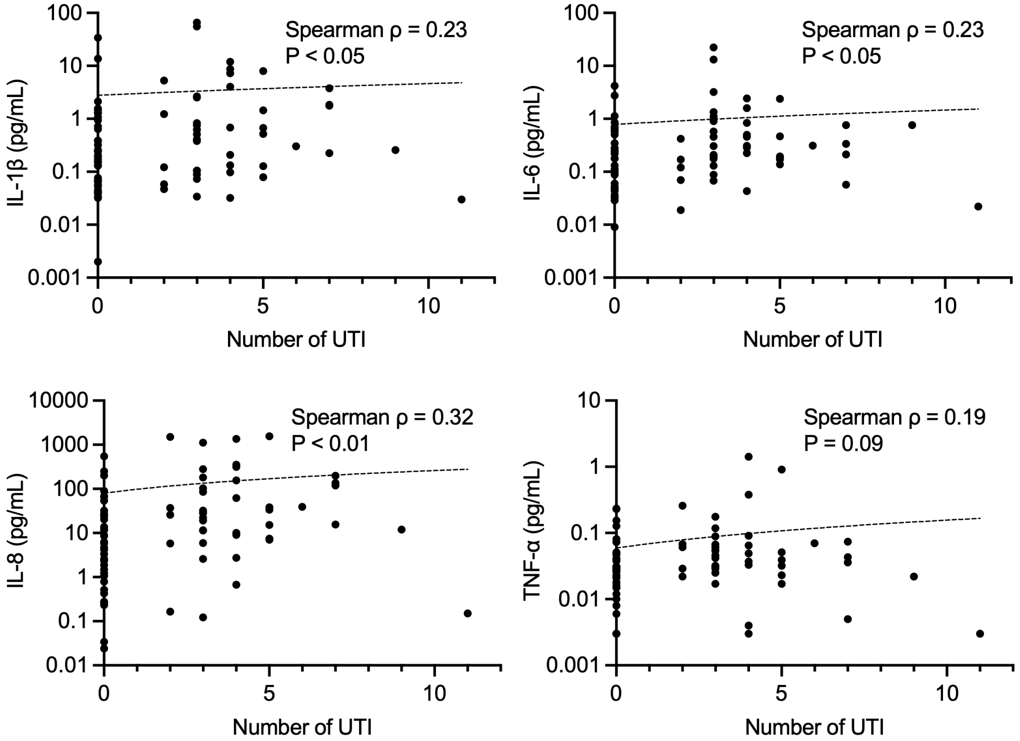

Supplemental Figure 6. Spearman correlation of urinary cytokines with number of prior UTI. Scatter plots depict the relationship between urinary cytokine concentrations and the number of prior UTIs across the entire study cohort. Spearman rank correlation coefficients (ρ) and corresponding two-tailed *P*-values are shown for each cytokine. IL-1β, IL-6, and IL-8 demonstrate modest positive correlations with increasing UTI burden, while TNFα shows a weaker, non-significant trend. Dotted lines indicate linear trend lines for visualization only.

SUPPLEMENTAL TABLE LEGENDS

Supplemental Table 1. Control and recurrent UTI study participant demographics and data. This information is included as an excel file.

Supplemental Table 2. Urine culture results in the recurrent UTI cohort. Each row lists the study number, age (years) at the time of each documented positive culture, and the urine culture result (colony forming units, organism isolated). Age in years was calculated from the date of birth and the culture date. Patients with multiple positive cultures are represented by multiple rows (one per culture). Not all clinically documented UTIs had available culture results; in such cases, rUTI classification and UTI counts were based on clinician documentation in the medical record. This information is included as an excel file.

Analyte	Controls < 8 years (*n*=11)	Controls ≥8 years (*n*=26)	*P*-value	rUTI < 8 years (n=16)	rUTI ≥8 years (n=26)	*P*-value
α-defensins 1-3 (pg/mL)	1397 (246.5-4535)	747.1 (253.6-1266)	0.32	3337 (2110-4753)	3770 (2884-4258)	0.89
β-defensin 1 (pg/mL)	554.4 (486.6-604.7)	477.5 (421.9-515.9)	0.02	389.2 (224.7-424.3)	406.6 (366.7-437.5)	0.15
Cathelicidin (ng/mL)	8.47 (6.45-10.34)	8.3 (7.49-10.51)	0.72	4.12 (3.26-6.02)	5.79 (3.51-8.18)	0.56
Lipocalin 2 (pg/mL)	430.8 (0.19-1380)	1167 (299.6-1726)	0.18	1457 (1106-2251)	1372 (591-2171)	0.73
RNase 7 (ng/mL)	372.9 (298.6-591)	747.1 (253.6-1266)	0.5	230 (152.3-774.4)	263.6 (170.5-723.4)	0.35
SLPI (pg/mL)	466.7 (309-731.5)	780.3 (291.8-866.8)	0.14	835.2 (669.6-1425)	1293 (753-1630)	0.16
IL-1β (pg/mL)	0.2 (0.13-1.27)	0.18 (0.05-0.71)	0.48	0.45 (0.07-1.69)	0.57 (0.12-2.93)	0.56
IL-6 (pg/mL)	0.18 (0.08-0.35)	0.16 (0.05-0.59)	0.77	0.17 (0.12-0.49)	0.32 (0.19-1.01)	0.09
IL-8 (pg/mL)	8.51 (1.22-25.33)	5.25 (0.72-33.81)	0.83	24.1 (6.69-104.9)	30.62 (9.42-166.3)	0.69
TNF-α (pg/mL)	0.03 (0.02- 0.04)	0.03 (0.02-0.04)	0.95	0.04 (0.03-0.07)	0.04 (0.02-0.07)	0.91

Supplemental Table 3. Urine analyte concentrations stratified by pubertal age group.

Values are presented as median (interquartile range. *P*-values were calculated using the Mann-Whitney *U* test.
